# O.4.6-5 Evaluation of physical activity policies in Scotland: results from the 2021 Active Healthy Kids Scotland Report Card

**DOI:** 10.1093/eurpub/ckad133.224

**Published:** 2023-09-11

**Authors:** Simone A Tomaz, John J Reilly, Avril Johnstone, Adrienne Hughes, Jenni Robertson, Leone C A Craig, Farid Bardid

**Affiliations:** University of Stirling, Stirling, UK; University of Strathclyde, Glasgow, UK; University of Glasgow, Glasgow, UK; University of Strathclyde, Glasgow, UK; Robert Gordon University, Aberdeen, UK; University of Aberdeen, Aberdeen, UK; simone.tomaz@stir.ac.uk; University of Aberdeen, Aberdeen, UK; simone.tomaz@stir.ac.uk; University of Strathclyde, Glasgow, UK

## Abstract

**Purpose:**

The benefits of physical activity (PA) are well established for children and adolescents, and national policies can play an important role in increasing child and adolescent PA. As part of the Active Healthy Kids Global Alliance initiative, the 2021 Active Healthy Kids Scotland Report Card summarised the status of PA and health in Scottish children and adolescents prior to the COVID-19 pandemic—reporting on a range of indicators (Bardid et al., 2022). This study provides a detailed examination of the evidence informing the Government and Policy indicator.

**Methods:**

Current Scottish PA policy documents (n = 18) published in 2011-2020—not including responses to COVID-19—were reviewed for grading. The grade was determined using an adapted version of the Policy Audit Tool version 2 (Ward et al., 2021). Key criteria in the scoring rubric include number and breadth of policies, identified funding, identifiable reporting structures, and monitoring and evaluation plan.

**Results:**

A C- grade was assigned to the Government and Policy indicator. There is clear evidence of leadership and commitment to increasing levels of PA and providing PA opportunities for children and adolescents. The allocation of funds and resources for implementation of policy has increased substantially since the publication of previous report cards. Progress through the key stages of public policymaking—policy agenda and formation—has improved. However, some policy documents do not identify accountable organisations, whilst others do not include details regarding reporting structures. Moreover, many policy documents do not provide information on monitoring and evaluation.

**Conclusions:**

Scotland has many creditable policies at national level. There appears to be good links between government directorates, policies and organisations responsible for implementation. Child and adolescent PA is clearly a priority in Scotland; it is not only an outcome, but also a means to achieve other goals (e.g., active travel to take climate action). However, current policies provide limited information on how delivery of proposed actions will be monitored and evaluated in practice. Future policies should therefore include more information on monitoring, evaluation, and reporting of delivery of actions, in order to better understand and support policy implementation and its impact on PA in children and adolescents.

